# A Comparative Study of Lidocaine and Lidocaine­ Mannitol in Anesthetizing Human Teeth with Inflamed Pulps

**Published:** 2006-04-01

**Authors:** Ali Talati, Maryam Bidar, Ghazal Sadeghi, Hossein Nezami

**Affiliations:** 1 *Department of Endodontics, School of Dentistry, Mashhad University of Medical Sciences, Mashhad, Iran*; 2 *Department of Endodontics, School of Dentistry, Tabriz University of Medical Sciences, Tabriz, Iran*

**Keywords:** Anesthesia, Lidocaine, Mannitol

## Abstract

**INTRODUCTION:** Failure to achieve adequate and profound anesthesia in teeth with acute pulp inflammation is a common condition during emergency visits in root canal therapy. Many different anesthetic solutions such as morphine and capsaicin have accordingly been examined. Mannitol­ an alcoholic sugar with high osmotic pressure level- is applicated for reducing intracranial and post retinal pressure in medicine. It has also been used for its diuretic effect. In combination with local anesthetic solution, it increases permeability of the nerve fiber sheath and leads to influx of the local anesthetic through cytoplasmic membrane .The purpose of the present study was to compare the efficacy of routine local anesthesia with or without using mannitol in teeth with inflamed pulps.

**MATERIALS AND METHODS:** one hundred patients with acute dental pain in posterior teeth were selected. Vials with 3 ml anesthetic solution containing 2.5% lidocaine with 1/80000 epinephrine or 2.5% lidocaine with 1/80000 epinephrine and 0.5 mol mannitol were used for anesthesia. For each patient, the routine injection technique was applied, during the removal of decay and dentine. Depth of anesthesia was evaluated and the supplementary injection was done in case of pain feeling and then pulpotomy was done. The analysis of data was done using chi-square statistical test.

**RESULTS:** The results showed that complete anesthesia after the first injection was obtained with lidocaine mannitol in 46% and with lidocaine alone in 38% of cases. However, the difference was not significant.

**CONCLUSION:** These finding suggest that the addition of mannitol to the standard anesthetic solution could insignificantly increase the level of anesthesia in teeth with inflamed pulps.

## INTRODUCTION

Failure to achieve adequate and profound anesthesia in teeth with acute pulp inflammation is a common condition during emergency visits in root canal therapy.

Malamed showed that acidic PH of an inflamed tissue can prevent anesthetic molecules entering the nerve sheath and leads to incomplete anesthesia. To obtain more anesthesia in such areas, volume of anesthesia molecules must be increased into the region (1).

Naggar proposed that inadequate pain control might be due to the fact that morphologic changes are developing and this morphologic changes in inflamed nerve even at a distance from the actual inflammatory site, is a significant barrier for normal electrolyte exchange at normal membrane level. Studies have demonstrated that inflammation potentiates peripheral nerve excitability (2,3).

Ghaziani et al. (4) showed that ability to anesthetize cats' inflamed teeth is less than normal teeth. Talati et al. (5) also showed that Topical use of capsaicin couldn’t lead to complete anesthesia in cat's teeth.

Mannitol- an alcoholic sugar with high osmotic pressure level- is applicated for reducing the intracranial and postretinal pressure in medicine, as well as diuretic effect. In combination with local anesthetic solution, it increases permeability of the nerve fiber sheath and leads to influx of the local anesthetic through cytoplasmic membrane (6). Because of its osmotic effect, mannitol is assumed to decrease cerebral edema. Mannitol might improve cerebral perfusion by decreasing viscosity, and as a free­ radical scavenger, it might act as a neuro protectant. Among its possible adverse effects, fluid and electrolyte imbalances cardiopulmonary edema, and rebound cerebral edema have been listed. It can activate the process of apoptotic cell death and has the potential to activate the inflammatory mediators that aggravate the neuronal injury due to ischemia (7).

Osmotic agents such as ionic hypertonic saline solution and nonionic mannitol, dextran, and lactose have been found to increase clearance of mucus and are regarded as promising mucoactive agents (8-11).

Reader (6) tested a composition of the local anesthetic lidocaine and mannitol. Alcohol opens protective coating of the sensory nerves allowing the anesthetic to enter the innermost parts of the nerves it is meant to numb. He tested the different versions of the formulation on more than 200 patients and found that about 90% of patients were able to achieve complete numbness, while, only 50% of patients with local anesthetic in the standard dosage experienced complete numbness.

The purpose of the present study was to compare the efficacy of routine local anesthesia with or without using mannitol in teeth with inflamed pulps.

## MATERIALS AND METHODS

For preparing local anesthetizing solution we used:

1- Lidocaine vials (Darupakhsh, Iran)

2- Epinephrine ampulles (Darupakhsh, Iran)

3- Mannitol serum 20% (Razy, Iran)

4- Lidocaine powder (Darupakhsh, Iran)

5- Distilled water

Vials of anesthetic solution were prepared in pharmacy school through two different Formulations:

Group A: 2.5% lidocaine + epinephrine 1/80000 + mannitol 0.5 molar

Group B: 2.5% lidocaine +epinephrine 1/80000 without mannitol

The solutions were prepared under Lamina flow Hood (with complete sterilization), then filtrated and sterilized using autoclave for 15 minutes.

The solutions were placed in 12 sterile 50 cc volume vials (6 vials containing mannitol and 6 vials without mannitol). They were then coded double blindedly and were kept at 37^°^c in microbial cultures for two weeks. None of the cultures showed microbial growth, which confirmed sterilization.

***Sample selection***

Patients between the ages of 18 to 60, referred to the endodontic clinic with acute dental pain, either in posterior maxillary or mandibular teeth, were selected as study population.

All of the patients were given a questionnaire to fill out and were carefully interviewed. Concerning their medical and dental history they were also asked about the duration, intensity and quality of dental pain and the time of their latest analgesic intake. The patients who had any systemic diseases or have had analgesic intake during the last 6 hour were excluded. Their chief complaint, subjective and objective signs and radiographs were studied and then vitality tests (electrical pulp test, heat tests) were done.

100 patients with diagnosis of irreversible pulpitis were finally selected. Pain intensity before treatment was assessed using 10 cm visual analog scale (VAS) ranging from no pain (0) to unbearable pain (10).

***Method of treatment***

For each patient we used one coded vial for anesthesia. First the code of via l was written in the patientʼs history sheet and then 3 cc of vial with a 5 cc syringe and needle gauge 25 was injected. The technique of injection for maxillary molars was supraperiosteal infiltration and in mandibular molars was inferior alveolar nerve block. In inferior alveolar nerve block, after 10 minutes of injection if the patient developed any signs and symptoms of anesthesia, cavity preparation was done and if the patient didnʼt feel pain, this was assumed as complete anesthesia after first injection. However, if the patient felt some pain, 1.5 cc from the same injection vial was injected and after 10 minutes cavity preparation was continued. If the patient didnʼt feel any pain, the case was labeled as complete anesthesia after second injection.

In case of pain at this point, the intensity of pain was recorded with VAS and then lidocaine 2% with epinephrine 1/80000 was injected using intrapulpal technique. Access preparation and pulpotomy was finally completed and the access cavity was sealed with cavit. The data were analyzed with the use of SPSS statistical software (chi-square test).

## RESULTS

The results showed that complete anesthesia after the first injection was obtained with lidocaine mannitol in 46% and with lidocaine alone in 38% of cases. However, the difference was not significant.

**Figure 1:**
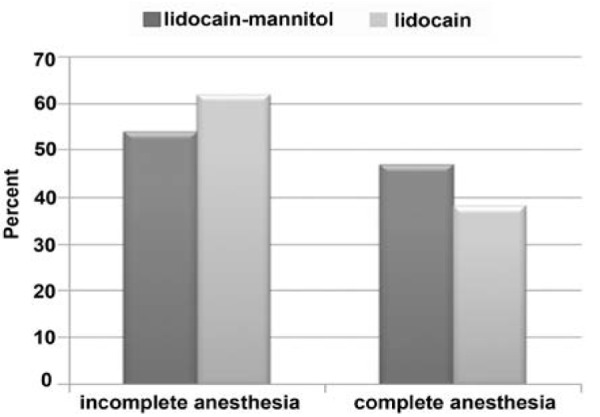
Frequency of patients with different success rates following the first injection

After second injection, complete anesthesia was obtained with lidocaine mannitol in 11.1% of cases and with lidocaine alone in 12.9%. Aga in the difference was not significant (Figure 1).

Cases with pre-treatment pain after cold test, lidocaine in 68.6% and lidocaine mannitol in 64.7% could not provide complete anesthesia but in cases without pain after cold test, complete anesthesia was not provided in 38.7%. In this case the difference was significant. In other words, statistical difference between pain after cold test and loss of complete anesthesia was significant.

The study also showed that complete anesthesia was achieved in teeth with mild pain in 66.7% of lidocaine group and 83.5% of lidocaine-mannitol group and totally 75%. Complete anesthesia, in teeth with moderate pain was obtained in 31.6% of lidocaine group and 55.6% of lidocaine­ mannitol group and totally 43.3%. Complete anesthesia in teeth with severe pain was obtained in 36% of lidocaine group and 30.8% of lidocaine-mannitol group and totally 33.35%.

**Figure 2:**
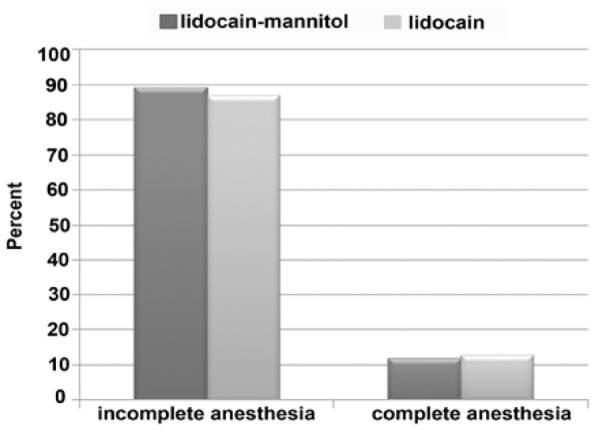
Incidence of complete and incomplete anesthesia in patients with mild, moderate and severe pain responses following cold test after first injection

**Figure 3:**
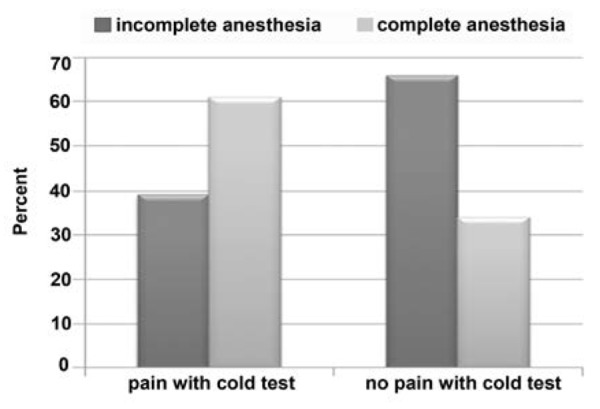
Incidence of complete and incomplete anesthesia in patients with different pain responses following the cold test

**Figure 4:**
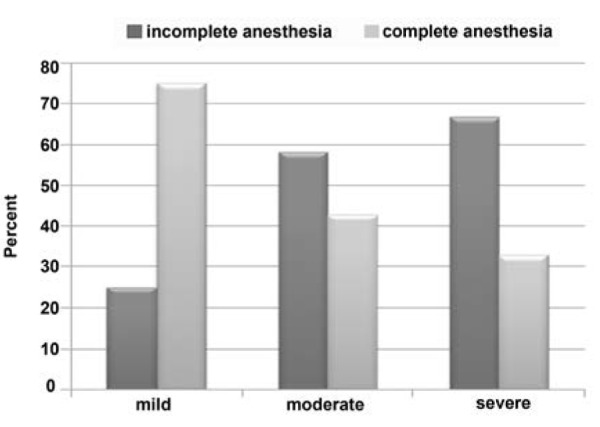
Incidence of complete and incomplete anesthesia in patients with mild, moderate and severe pain responses following cold test after the first injection

Thus the difference in the rate of complete anesthesia among the teeth with different pain intensities was significant (p<0.05) (Figure 2), (Figure 3), (Figure 4).

## DISCUSSION

In this study mannitol was used for increasing lidocaine anesthetizing effects in teeth with inflamed pulps. The aim of this study was to compare the efficacy of routine local anesthesia with and without mannitol in teeth with pulpitis. Mannitol in combination with local anesthetic solution increases permeability of the nerve fiber sheath and leads to influx of the local anesthetic through cytoplasmic membrane. Reader (6) used a certain composition of local anesthetic lidocaine and mannitol for anesthetizing teeth with inflamed pulps.

He tested different samples of the formulation on more than 200 patients. Reader found that best results occurred when a mixture of mannitol and approximately 64 mg of lidocaine was used. He also found that about 90% of patients experienced complete numbness, while only 50% of patients for whom only local anesthetic agent in the standard dosage was used experienced such numbness. He showed that mannitol with 75% concentration or 0.9 molar is irritant and create trismus after injection but 0.5 molar concentrations is less irritant and has more efficacy. Lidocaine with a concentration more than 2.5% is also irritant and causes trismus and pain.

In this study we used 2 groups with equal amounts of lidocaine: Group A: 2.5% lidocaine + epinephrine 1/80000 +mannitol 0.5 molar and Group 8: 2.5% lidocaine + epinephrine 1/80000 without Mannitol. These two groups were applied double-blindly (each group for 50 patients). Our study showed that there is no significant difference for different ages and sexes between 2 groups. It also showed that complete anesthesia after the first injection was achieved in 38% of group B and in 46% of group A. However, in Reader’s study inject ion of lidocaine mannitol in 90% of cases showed success. This result might be due to difference in sampling of the anesthetic solution.

Ghaziani showed that lidocaine alone could create complete anesthesia after the first injection in 20% of the cases which is different from our study (38%) (4). This may be because of increase in lidocaine volume (2.5% and 3cc) in our study. Malamed and Weine showed that in inflammatory conditions, increase in the volume of anesthetic solution is more efficient than application of an agent with a greater potency (1). Our study also showed that lidocaine could develop complete anesthesia in 36.7% of cases in mandibul and in 40% of cases in maxilla after the first injection. These rates were 48.3% and 42.9% for lidocaine-mannitol group respectively. However, the difference was not significant. Malamed showed that anesthetizing mandibular molars is more difficult than other teeth. In this study, the use of lidocaine­ mannitol solution in mandibular molars showed more success than lidocaine alone. It also showed that in second injection, in 11.1% of group A and 12.9% of group B complete anesthesia was obtained, thus for anesthetizing teeth with inflamed pulps, we can increase the dosage of lidocaine solution. If complete anesthesia did not occur, other supplementary techniques could be used.

Results of our study showed that complete anesthesia was obtained in teeth with mild pain in 75% of the total study population, 41.3% having teeth with moderate pain and 33.35% having teeth with severe pain the differences were statistically significant. Thus we conclude that with increase of pain intensity, the effectiveness of anesthetic solution was decreased.

In cases that the tooth was sensitive to cold test, complete anesthesia was obtained in 33.2% of total population but in non-sensitive cases 61.3% showed complete anesthesia. The difference was statistically significant.

Considering the lower response of the mandible to anesthesia and 12% difference in the two groups, other studies concentrating on the lower jaw and also histochemically assays on mannitol in nerve cell is recommended.
